# Editorial: Reproductive Neuroendocrinology and Social Behavior

**DOI:** 10.3389/fnins.2016.00124

**Published:** 2016-04-13

**Authors:** Ishwar S. Parhar, Tomoko Soga, Sonoko Ogawa

**Affiliations:** ^1^Brain Research Institute, School of Medicine and Health Sciences, Monash UniversityKuala Lumpur, Malaysia; ^2^Laboratory of Behavioral Neuroendocrinology, University of TsukubaTsukuba, Japan

**Keywords:** HPG axis, social bonding, aggression, GnRH, oxytocin vasopressin, neurotransmitter, sex steroids

Reproduction consists of various physiological events including fertilization, development of sexual characteristics, social behavior, maturation, and aging. Reproductive functions are ultimately regulated by the hypothalamus-pituitary-gonadal (HPG) axis. Gonadotropin-releasing hormone (GnRH) is a pivotal hypothalamic neuropeptide that regulates vertebrate reproduction (Schally et al., 1972). In tetrapods, GnRH neurons are located in the preoptic-hypothalamic region and project their fibers to the median eminence to regulate gonadotropin secretion from the anterior pituitary gland, which stimulates sex steroid secretion and gametogenesis in the gonads. It was also shown that central administration of GnRH can stimulate female sexual behavior in rats (Moss and McCann, 1973; Pfaff, 1973). GnRH release is regulated by other neuropeptides, neurotransmitters, and steroid hormones. Watanabe et al. summarize the role of gamma-amino butyric acid (GABA) in the regulation of GnRH neuronal activity and discuss functional consequences of GABAergic inputs to GnRH neurons in physiological aspects of reproduction. Recently, two neuropeptides containing the C-terminal Arg-Phe-NH_2_ (RFamide peptides), kisspeptin, and gonadotropin-inhibitory hormone (GnIH), emerged as critical accelerator, and suppressor, respectively, of vertebrate reproduction. Parhar et al. highlight classical and recent findings regarding the role of GnRH, kisspeptin, and GnIH in the regulation of social behaviors in fish, birds, and mammals, and discuss their importance in future biological and biomedical researches (Perspectives).

As social behaviors such as courtship, mating, and aggression are strongly associated with sex steroids (Adkins-Regan, 2005), hypothalamic neuropeptides can regulate social behaviors by regulating the HPG axis. It was originally thought that males display male-typical behaviors because they are exposed to androgen and females display female-typical behaviors because they are exposed to estrogen or progestogen. However, it was later discovered that central actions of androgen in males for the expression of certain male-typical behaviors require its aromatization into neuroestrogen (aromatization hypothesis; Yahr, 1979). Ubuka and Tsutsui summarize investigations on how aromatase expression and activity are regulated in the brain and discuss how neuroestrogen regulates socio-sexual behavior of males. Change in androgen concentration in response to social challenges has been hypothesized as one of the regulatory mechanisms of behavior in response to the perceived social environment (Challenge hypothesis, Wingfield et al., 1990). Oliveira and Oliveira review studies on the mechanism and function of androgen response to social challenges and discuss the modulatory mechanism of social decision-making by peripheral hormones. Sakuma summarizes detailed mechanisms of estrogen-sensitive preoptic area (POA) neurons regulating sexual behavior of female rats. According to Sakuma, there are separate subpopulations of POA neurons, facilitatory proceptive components of female sexual behavior such as copulation solicitation, and inhibitory receptive components of female sexual behavior such as lordosis reflex. POA neurons controlling proceptivity are estradiol benzoate (EB)-sensitive and project to the midbrain locomotor region. EB-sensitive projections of POA neurons to the ventral tegmental area (VTA) controls lordosis reflex. EB disinhibits lordosis reflex by inhibiting POA neurons innervating the VTA that innervates medullospinal neurons innervating spinal motor neurons of the back muscle (Sakuma). Tsukahara and co-workers introduce lateral septum (LS) neurons inhibiting lordosis in both female and male rats, and discuss the neuroanatomy and sex differences of the lordosis-inhibiting system in the LS. Neurons of the intermediate part of the LS (LSi) inhibits lordosis by projecting axons to the midbrain central gray (MCG). The LSi-MCG neural connection is sexually dimorphic, and formation of the male-like LSi-MCG neural connection is affected by aromatized testosterone in the postnatal period (Tsukahara et al.). Lordosis is induced by a male mount stimulus when the female has proestrus levels of estrogen. The mount stimulus signal is conveyed through the anterolateral column of the spinal cord and sent to the medullary reticular formation (MRF) and periaqueductal gray (PAG; Pfaff, 1980). The ventromedial nucleus of the hypothalamus (VMH) is the major site of estrogen action for the induction of lordosis (Rubin and Barfield, 1983). Estrogen receptor (ER)-expressing neurons in the VMH project to the PAG (Calizo and Flanagan-Cato, 2003). These results imply that PAG neurons stimulated by a mating stimulus induce lordosis, however the neurotransmitter in PAG neurons projecting to the MRF is still unknown. Yamada and Kawata present original findings suggesting that glutamatergic neurons in the lateral PAG that project to the MRF are involved in lordosis behavior of female rats.

Various effects of estrogen are mediated by the nuclear receptors, ERα and ERβ (Green et al., 1986; Kuiper et al., 1996; Ogawa et al., 2000). Matsuda summarizes epigenetic changes in the ERα gene promoter, such as histone modifications and DNA methylation, which appear to be crucial for determining the extent of socio-sexual behaviors between the sexes and among individuals within the same sex. Brain areas such as the bed nucleus of the stria terminalis, amygdala, medial preoptic nucleus, dorsal raphe nucleus, and locus coeruleus express both ERs, but the supraoptic nucleus (SON) and paraventricular nucleus of the hypothalamus (PVN) exclusively express ERβ (Shughrue et al., 1996; Mitra et al., 2003). Specific expression of ERβ in the SON and PVN suggests potential involvement of ERβ in the regulation of anxiety-related social behaviors as well as stress responses (Handa et al., 2012). Maternal separation (MS) is known to severely affect social and anxiety behaviors in mice (Tsuda and Ogawa, 2012). Tsuda and co-workers investigate whether ERβ mediates the effect of MS stress on these behavioral alterations using ERβ knockout (βERKO) mice. βERKO mice are still sensitive to MS effects on female and male social behaviors, suggesting that MS overrides ERβ effects on female social anxiety and male aggression (Tsuda et al.). Diethylstilbestrol (DES) is an active synthetic non-steroidal estrogen, which is widely used as a model chemical to study the effects of estrogenic endocrine disruptors on both the physical and behavioral development of offspring. Tomihara and co-workers orally administer DES to pregnant female mice and investigate the maternal behavior of mothers. They also examine the direct effects of DES exposure *in utero* as well as the indirect effects of aberrant maternal behavior on the offspring by cross-fostering method. The results show the risks of endocrine disruptors on the mother and the offspring, suggesting that developmental deficits of offspring may stem from both *in utero* toxicity and aberrant maternal care (Tomihara et al.). 17α-ethinyleestradiol (EE2) is a potent estrogenic compound which is mainly used as oral contraceptives. Derouiche and co-workers investigate its effects on the reproductive function of female mice that were exposed to EE2 during development. Their results put emphasis on the high sensitivity of sexual dimorphic behaviors and neuroendocrine circuits to disruptive effects of endocrine disrupting chemicals (Derouiche et al.).

Sex-specific behavior and brain structure have been thought to be shaped by perinatal sex steroids secreted by the gonads. However, recent studies on the sex-determining gene in mammals and gynandromorphic birds have suggested the sex chromosomal effects on sex differences in aggression levels and social interaction. Maekawa et al. summarize current understandings of the roles of sex steroids and sex chromosomes in the determination of brain related to sexual behavior and reproduction in mammals and birds. A sex changing fish, bi-directionally hermaphroditic *Lythrypnus dalli*, is an excellent model for a deeper understanding of fitness associated with behavior and the endocrine system. Pradhan et al. propose that local steroids regulation is one possible mechanism that allows for the expression of novel phenotypes that characterizes specific life history stages. Sakamoto introduces that spinal cord contains several neural circuits, showing a clear sexually dimorphism, which are critical in expressing penile reflexes and discusses the functional and anatomical significance of the sexually dimorphic nuclei in the spinal cord in relation to the expression of male sexual behavior. There are also sex differences in the feeding system and responses to fasting, sex steroids, and diet. Fukushima et al. explain that melanin-concentrating hormone and orexin neurons in the lateral hypothalamic area are the key systems that play significant roles in making sex differences in feeding behavior.

Oxytocin (OXT) is a nine-amino acid neuropeptide that was discovered in 1906 as having uterus contracting effects (Dale, 1906), and it was the first peptide hormone to be sequenced (du Vigneaud et al., 1953). OXT is primarily synthesized in magnocellular neurosecretory cells in the PVN and SON, projecting their axon terminals into the posterior pituitary, where it is released into the general circulation. OXT is well known for its role in milk ejection reflex and it is also involved in the regulation of behaviors, such as social recognition, anxiety, feeding, anti-nociception, and stress responses. Hashimoto et al. review their studies that visualized OXT by fusion of fluorescent protein gene in the hypothalamo-neurohypophysial system of rats. Arginine vasopressin (AVP) is structurally similar to OXT, and these neuropeptides are involved in the regulation of social behaviors including pair bonding, parental behavior, affiliation, and aggression. Lieberwirth and Wang review the roles of OXT and AVP in social bonding in mammals including humans, and discuss the roles of OXT and AVP in social bond formation between mating pairs as well as parents and their offspring, and the formation of interpersonal bonding involving trust. It has also been suggested that OXT affects processing of infant face sight and emotional reaction to infants. Saito and co-workers show that urinary OXT positively correlates with facial visual search task performance in unmarried healthy male. However, task performance and its correlation with OXT concentration were the same between infant faces and adult faces. Their results suggest that endogenous OXT is related to facial cognition, although OXT is not related to infant-specific responses in unmarried men (Saito et al.). Fujisawa and co-workers investigate the relationship between visual attention for social information and OTX levels in Japanese preschool children with autism spectrum disorder (ASD). They measure salivary OXT levels and the pattern of gaze fixation for social information. There is a positive association between salivary OXT levels and gaze fixation duration to an indicated object area in typically developing children. However, no association is found between these variables in children with ASD (Fujisawa et al.). The nanopeptide vasotocin (VT) is a non-mammalian homolog of mammalian OXT or vasopressin, which influences a variety of sex-typical and species-specific behaviors. Kelly and Goodson quantify social contact and anxiety-like behavior after bilateral antisense knockdown of VT production in the medial bed nucleus of the stria terminalis (BSTm) of male and female Angolan blue waxbills. They show that BSTm VT neurons promote social contact, not gregariousness, and that the effects of antisense on social contact are stronger in male birds than in females (Kelly and Goodson).

Interaction between male and female is important to find mating partners and for reproductive success. Male sexual signals such as pheromones transmit information and social and sexual status to females. Male vocalizations also enhance reproductive function in females. Asaba et al. summarize the effects of olfactory and auditory cues on the behavior and neuroendocrine functions of females, and discuss how male signals are processed in the brain to regulate the reproductive function and behavior of females. Lado and co-workers use male goldfish forebrain explants *in vitro* and perform whole-cell current clamp recordings from single neurons in the ventral preoptic area (vPOA) to characterize their membrane properties and synaptic inputs from the olfactory bulbs (OB). Data from electrical stimulation of the OB and application of receptor antagonists suggest that vPOA neurons receive monosynaptic glutamatergic inputs via the medial olfactory tract, with connectivity varying among neuronal groups (Lado et al.). Sex pheromones from ovulatory females stimulate male sexual behavior, such as chasing, and sperm releasing act in goldfish. Kawaguchi and co-workers examine the involvement of olfaction in the sexual behavior of goldfish. No behavior is elicited in males without olfaction and pheromonal stimulation. The lack of olfaction inhibits sexual behavior in females mediated by the olfactory pathway. Their results show that regulation of sexual behavior of goldfish has gender-typical olfactory mechanism (Kawaguchi et al.).

Paternal behavior is not well understood compared to maternal behavior. Liu et al. (2013) previously reported that male ICR strain laboratory mice can display maternal-like parental care (pup retrieval) by signals from the pair mate. Liang and co-workers report in this research topic that the pair mate-dependent paternal retrieval behavior is observed in the ICR strain but not in C57BL/6 or BALB/c mice. ICR sires display retrieval behavior only to his biological pups. Their results indicate that the ICR sires display unique paternity (Liang et al.). Many studies have shown that daily repeated MS stress can regulate the hypothalamic-pituitary-adrenal (HPA) axis and affect subsequent brain function and behavior during adulthood, although the molecular basis of the long-lasting effects of early life stress on brain function is not fully elucidated. Nishi and co-workers present various cases of MS in rodents and illustrate alterations in the HPA axis activity. They also characterize the brain regions affected by various patterns of MS, including repeated MS and single time MS at various stages before weaning, by investigating c-Fos expression. They emphasize how early life stress can affect behaviors, by inducing depression, anxiety, or eating disorders, and alters gene expression in MS adult mice (Nishi et al.). Recent study has shown that post-weaning social isolation stress induces symptoms of *depression* and anxiety and decreases expression of reproductive neuropeptides such as GnRH and GnIH in male rats (Soga et al.). The environmental factor related parental care during the pre- and post-pubertal period may also be crucial to control social and emotional behavior and reproduction. Affective responses to mother, an attachment figure, may change during puberty in boys. Takamura and co-workers compare the neural response of boys to visual images of their own mothers at three different developmental stages throughout puberty. They measure their neural response in the anterior part of the prefrontal cortex (APFC) to their mother's smiling face compared with that of an unfamiliar-mother. Their findings suggest that different patterns of APFC activation are associated with changes in response to the mother in puberty (Takamura et al.).

Perceptions of the dominance level of themselves and others, and the ability to control their behavior adequately according to the dominance levels are crucial for living within a social environment. Watanabe and Yamamoto review investigations of neural substances that are involved in the perception of social dominance and the formation of social hierarchy by recent brain imaging and molecular techniques. Dominant and subordinate dispositions are not only determined genetically but also nurtured by environmental stimuli during neuroendocrine development, although the relationship between early life environment and dominance behavior remains elusive. Benner and co-worker review two cases in which environmental insults during the developmental period alter the outcome of dominance behavior later in life. Similar alterations are found in the cortex and limbic area in mice that were isolated from their mother and their littermates, and mice that were perinatally exposed to a pollutant, suggesting that the neural systems are shared in dominance behavior (Benner et al.). Aggression is one of the common social behaviors that are observed in the animal kingdom, and the involvement of serotonin system in the control of aggressive behavior has been confirmed (Olivier et al., 1995). Takahashi and co-workers show that the Japanese wild-derived mouse strain MSM/Ms (MSM) retains higher level of aggression than the laboratory strain, C57BL/6J. They further analyze the genetic and neurobiological mechanism in different strains and find that *Tph2*, a gene encoding an enzyme involved in serotonin synthesis in the midbrain is increased in chromosome 4 consomic strain and MSM, and that there is a positive genetic correlation between aggressive behavior and *Tph2* mRNA expression (Takahashi et al.).

Most vertebrates living in the temperate zone show physiological and behavioral responses to seasonal changes in photoperiod. Nakane and Yoshimura introduce and discuss the photoperiodic signal transduction pathways that may regulate seasonal reproduction in birds, mammals and fish. Melatonin is produced mainly in the pineal gland and retina in vertebrates, and its concentration is higher during night than day-time. This daily rhythm of circulating melatonin informs the organism about the time within a day, whereas the duration of the nocturnal elevation of melatonin that corresponds to photoperiod informs the organism about the season within a year (Reiter, 1993). Ikegami and co-workers examine melatonin receptor gene expression as well as melatonin synthesis and secretion in the pineal gland of grass puffer that shows unique lunar/tidal cycle-synchronized mass spawning. Their results suggest the importance of cyclic melatonin receptor gene expressions in the pineal gland in the control of the lunar/tidal cycle-synchronized mass spawning of grass puffer (Ikegami et al.). Impairment of neural functions occurs frequently when aquatic vertebrates, particularly fish, are exposed to low oxygen (Thomas and Rahman, 2009). Tryptophan hydroxylase (TPH), involved in serotonin synthesis, is a neuroenzyme liable to oxygen. Accordingly, maintenance of oxygen levels is essential to maintain its enzymatic activity (Kuhn et al., 1980). Rahman and Thomas investigate if antioxidant treatment prevents hypoxia-induced down-regulation of hypothalamic TPH and serotonergic functions in Atlantic croaker. Their results suggest that hypoxia-induced reduction of TPH and serotonergic functions are mediated by neuronal nitric oxide synthase, and generation of free radicals and a decrease in the antioxidant status are involved (Rahman and Thomas).

Collectively, all articles contained in this research topic provide classical knowledge in reproductive neuroendocrinology and social behavior, and also most updated knowledge in all aspects of neurobiological mechanisms regulating social behavior in vertebrates.

## Author contributions

All authors listed, have made direct contribution to the writing, and approved it for publication.

### Conflict of interest statement

The authors declare that the research was conducted in the absence of any commercial or financial relationships that could be construed as a potential conflict of interest.
